# Quantification of Dialytic Removal and Extracellular Calcium Mass Balance during a Weekly Cycle of Hemodialysis

**DOI:** 10.1371/journal.pone.0153285

**Published:** 2016-04-13

**Authors:** Jacek Waniewski, Malgorzata Debowska, Alicja Wojcik-Zaluska, Andrzej Ksiazek, Wojciech Zaluska

**Affiliations:** 1 Department for Mathematical Modelling of Physiological Processes, Institute of Biocybernetics and Biomedical Engineering, Polish Academy of Sciences, Warsaw, Poland; 2 Department of Physical Therapy and Rehabilitation, Medical University of Lublin, Lublin, Poland; 3 Department of Nephrology, Medical University of Lublin, Lublin, Poland; Emory University, UNITED STATES

## Abstract

**Objectives:**

The removal of calcium during hemodialysis with low calcium concentration in dialysis fluid is generally slow, and the net absorption of calcium from dialysis fluid is often reported. The details of the calcium transport process during dialysis and calcium mass balance in the extracellular fluid, however, have not been fully studied.

**Methods:**

Weekly cycle of three dialysis sessions with interdialytic breaks of 2-2-3 days was monitored in 25 stable patients on maintenance hemodialysis with calcium concentration in dialysis fluid of 1.35 mmol/L. Total and ionic calcium were frequently measured in blood and dialysate. The volume of fluid compartments was measured by bioimpedance.

**Results:**

Weekly dialytic removal of 12.79 ± 8.71 mmol calcium was found in 17 patients, whereas 9.48 ± 8.07 mmol calcium was absorbed per week from dialysis fluid in 8 patients. Ionic calcium was generally absorbed from dialysis fluid, whereas complexed calcium (the difference of total and ionic calcium in dialysis fluid) was removed from the body. The concentration of total calcium in plasma increased slightly during dialysis. The mass of total and ionic calcium in extracellular fluid decreased during dialysis in patients with the dialytic removal of calcium from the body and did not change in patients with the absorption of calcium from dialysis fluid.

**Conclusions:**

We conclude that about one third of patients on dialysis with calcium 1.35 mmol/L in dialysis fluid may absorb calcium from dialysis fluid and therefore individual prescriptions of calcium concentration in dialysis fluid should be considered for such patients.

## Introduction

Mineral metabolism in patients with chronic kidney disease is often disturbed with bone disorders and soft tissue calcification [1]. Mineral indices in these patients are related to mortality and morbidity [2–4]. The mass balance of calcium in patients on hemodialysis (HD) has been controversial for a long time [5–7]. It can be controlled to some extent by adjusting calcium concentration in dialysis fluid [8–14]. The modification of calcium concentration in fresh dialysis fluid induces changes not only in calcium levels in plasma but also in plasma levels of phosphate and parathyroid hormone [15]. Different concentrations were proposed and the calcium mass balance was investigated in clinical studies with concentration between 1.25 and 1.5 mmol/L as the currently accepted concentration range [1]. Basile et al. indicated that dialysis fluid of total calcium concentration in the middle of 1.25 and 1.5 mmol/L might be preferable because it gives a mildly positive calcium mass balance, maintains normal serum calcium level and does not stimulate short-term parathyroid hormone secretion during dialysis [16]. With this concentration the calcium intake from dialysis fluid, although still observed, is mostly avoided or kept low while the appreciable decrease of calcium concentration in plasma that may yield arrhythmia does not occur [9]. However, the details of calcium transport during HD and the changes of calcium concentration and mass in extracellular compartment during and between dialysis sessions for the whole week of standard dialysis cycle have not been fully investigated.

The problem of quantification of calcium balance during dialysis sessions is complicated by the occurrence of different forms of calcium in plasma and dialysate [17]. The concentration of total calcium is typically measured in plasma and ionic calcium can also be assayed by ion selective electrode. However, the third form of calcium–calcium ions complexed with small anions–is more difficult to assess [18]. Nevertheless, complexed calcium plays an important role in the mass balance of calcium during dialysis, as we show in the present study.

Dialysis is one of a few factors that contribute to the control of calcium level in plasma. The other factors include the exchange with bone compartment, calcium intake from the gastrointestinal tract, reabsorption in the kidneys (in patients with residual renal function) as well as short and long term hormonal control of these processes [4, 17]. Therefore, the analysis of the changes in the concentration of different forms of calcium in plasma (extracellular fluid) throughout the whole standard weekly cycle of three dialysis sessions may help to understand the effect of dialysis on this physiologically important parameter. Furthermore, the assessment of extracellular water volume and its changes throughout the cycle of dialysis by bioimpedance allows to describe the changes in extracellular store of calcium.

We present a quantitative assessment of calcium kinetics and mass balance during the weekly cycle of three HD sessions with low (1.35 mmol/L) calcium concentration in dialysis fluid and propose to apply *equivalent continuous clearance*, *ECC*, to quantify the removal of calcium by HD. The calcium mass balance during HD and the effect of different transport mechanisms in dialyzer on calcium removal or absorption were recently debated [19]. However, the role of two different forms of calcium in dialysis fluid: 1) ionic and 2) diffusible but complexed to small anions–in net calcium balance was not assessed yet. We also present a quantitative evaluation of calcium kinetics and mass balance in extracellular compartment during the weekly cycle of three HD sessions.

## Methods

### Patients and clinical data

Three consecutive HD sessions of one week dialysis treatment cycle with the interdialytic breaks of 2-2-3 days were evaluated in 25 anuric patients (16 females, age 62.3 ± 14.0 year, body weight 67.8 ± 15.5 kg, body mass index 24.5 ± 4.4 kg/m^2^) [20, 21]. The study was approved by the Bioethical Committee at the Medical University of Lublin (Poland) and written informed consent was obtained from each patient.

HD treatments were performed using high flux dialyzers (4 patients) or low flux dialyzers (21 patients) with dialysate inflow 500 mL/min. The blood flows varied between patients (200–350 mL/min) but were constant for the patient throughout the three consecutive HD sessions. Ultrafiltration was calculated based on the weight drop corrected (if necessary) for fluid infused and/or drunk by patient during dialysis. The session time was fixed to four hours for each patient and was on average 239 ± 11 min. The concentration of calcium in bicarbonate buffered dialysis fluid was 1.35 mmol/L. Concentrations of total (by calorimetric method with Arsenazo, Advia 1800, Siemens) and ionized (by ion selective electrode and potentiometry indirect mode, RapidLab 348, Siemens) calcium were measured in serum before, at 1, 2 and 3 h, at the end and 45 min after each session, before the fourth dialysis session and in the outlet dialysate every 0.5 h. The calcium concentration in the inlet dialysis fluid was measured at 2 h of dialysis. In a separate previous investigation with frequent measurements of this concentration at 5, 30, 60, 90, 120, 150, 180, 210 and 240 min of dialysis in 44 dialysis sessions for 11 patients we found no statistically significant difference between the measured values for different sampling times and, in particular, no difference between the values at any one sampling time vs. the average value per session (data not shown). During the monitored HD sessions the level of parathyroid hormone (PTH 1–84) was assayed once using enzyme-linked immunosorbent assay (ELISA kit, Immutopics, Inc., San Clemente, CA, USA). The volume of total and extracellular fluids were assessed by bioimpedance (Body Composition Monitor, Fresenius Medical Care, Bad Homburg, Germany) before, at the end, and 45 min after each session.

### Calculations

The removed mass of calcium was calculated from the flow rate of dialysate and concentrations at inlet and outlet of dialyzer. The contribution of ultrafiltration to calcium transport was evaluated as ultrafiltration rate multiplied by calcium concentration in outlet dialysate.

The mass transported by diffusion, *ΔM*_*R*,*Diff*_, was calculated as the difference between the total removed mass, *ΔM*_*R*_, and the mass removed by ultrafiltration, *ΔM*_*R*,*UF*_ (*ΔM*_*R*,*Diff*_ *= ΔM*_*R*_*-ΔM*_*R*,*UF*_).

To calculate the total calcium ion concentration in plasma water from the diffusible ion concentration measured by direct electrode mode in serum, the measured value was multiplied by 1.067/DF, where DF = 0.96^2^ is the Donnan factor for bivalent cation and the factor 1.067 is to take into account the protein and lipid content of serum [22]. The concentration and mass of complexed calcium in dialysis fluid was calculated as the difference between the concentration and mass of total calcium and concentration and mass of ionized calcium, respectively.

Dialytic *equivalent continuous clearance*, *ECC*, was calculated as the removed mass, *ΔM*_*R*_, per dialysis cycle time (*T*_*c*_ *= one week*) per the time average solute concentration, $\bar{C_{b}}$, in serum, $ECC=\Delta M_{R}/\left(T_{c}\cdot \bar{C_{b}}\right)$, [23, 24]. The average values of total and ionic calcium concentration in serum were calculated from the weekly profiles of calcium in serum. The concentration of complexed calcium in serum could not be measured and therefore it was assumed 0.37 mmol/L and constant, according to the data for healthy subjects [18] and applied in some estimations of transport characteristics of complexed calcium for tentative comparison with ionic calcium.

### Statistical analysis

The data are presented as mean ± standard deviation (SD). The comparison between calcium concentration at inlet and outlet of dialyzer was performed using two-sided rank sum test. Kruskal-Wallis test and multiple comparison procedure were used whenever appropriate. Statistical dependence between variables was measured by Spearman's correlation coefficient (*rho*). Statistical significance was set at the level of *p-value <* 0.05, unless otherwise indicated.

## Results

The measured concentration of total calcium in inlet dialysis fluid was 1.35 ± 0.12 mmol/L and in the outlet dialysate 1.32 ± 0.16 mmol/L (p = 0.03), Table 1. The concentration in dialysis fluid of ionized calcium significantly decreased and complexed calcium significantly increased from inlet to outlet of dialyzer, Table 1. Blood serum concentration of PTH 1–84 was 295.5 ± 379.5 pg/mL.

**Table 1:** Concentration of calcium (total, ionized and complexed, mmol/L) in dialysis fluid in the inlet and outlet of dialyzer in 75 dialysis sessions (at 2 hour), mean ± SD.

Ca concentration mmol/L	Dialyzer inlet	Dialyzer outlet	Inlet vs. outlet
Total	1.35 ± 0.12	1.32 ± 0.16	p = 0.03
Ionized	1.06 ± 0.09	1.01 ± 0.08	p < 0.001
Complexed	0.28 ± 0.07	0.32 ± 0.13	p < 0.001

As demonstrated by the mass balance in dialysis fluid, the total and complexed calcium was on average removed from the body in most sessions, however ionized calcium was on average absorbed from dialysis fluid, Table 2. The removal of calcium from the body was observed in 50 dialysis sessions, whereas in 25 sessions calcium was absorbed from dialysate. The removal of ionized and complexed calcium was by convection and the complexed calcium was removed also by diffusion, Table 2. The convective removal of total, ionic and complexed calcium was higher during the sessions after the long interdialytic break with higher ultrafiltration than during sessions after 2-day break (ultrafiltration equal 2.99 ± 0.82 L and 2.19 ± 0.76 L after 3-day and 2-day break, respectively, *p-value <* 0.001), Table 2. No statistically significant difference between the sessions after long and short breaks was found for overall calcium removal; the mass of all calcium forms removed by diffusion was not different either, Table 2. Although the mean calcium removal during 4 h dialysis session was not high, there was high inter-session variation as demonstrated by high SD values, Table 2, and high scattering of the mass removed per session with ranges from -18.4 to 17.6 mmol for total calcium, from -20.6 to 5.4 mmol for ionized calcium, and from -14.8 to 27.5 mmol for complexed calcium per session (negative sign means calcium absorption from dialysis fluid to the body).

**Table 2:** Mass of total, ionized and complexed calcium (in mmol) removed or absorbed (negative sign) from/into patient body by convective, diffusive and overall transport in 75 dialysis sessions and separately for sessions after 3- and 2-day break, mean ± SD.

Removed mass of Ca, mmol	Overall	By convection	By diffusion
*All sessions*, *N = 75*			
Total	1.89 ± 6.32	3.22 ± 1.18	-1.33 ± 6.37
Ionized	-4.51 ± 4.76	2.41 ± 0.87	-6.92 ± 4.85
Complexed	6.40 ± 6.57	0.80 ± 0.35	5.60 ± 6.51
*Sessions after 3-day break*, *N = 25*			
Total	2.20 ± 4.30	3.95 ± 1.16	-1.75 ± 4.50
Ionized	-3.16 ± 4.30	2.97 ± 0.88	-6.13 ± 4.47
Complexed	5.36 ± 3.59	0.98 ± 0.34	4.38 ± 3.63
*Sessions after 2-day break*, *N = 50*			
Total	1.73 ± 7.15	2.85 ± 1.02*	-1.11 ± 7.15
Ionized	-5.18 ± 4.88	2.14 ± 0.73*	-7.32 ± 5.03
Complexed	6.92 ± 7.62	0.71 ± 0.32*	6.20 ± 7.51

* statistically significant difference vs. sessions after 3-day break.

The *equivalent continuous clearance*, *ECC*, for calcium was generally low and negative for ionized calcium (because of its absorption to the body instead of removal), and positive for complexed calcium, Table 3. ECC of ionized calcium had the value similar, but of opposite sign, to the kidney clearance of ionic calcium in normal individuals of 1.5 mL/min [17]. Therefore, because ionic calcium was absorbed to the body, ECC of total calcium was three times lower in patients with weekly dialytic removal of calcium (ECC = 0.56 ± 0.38 mL/min, Table 3) and six times lower (ECC = 0.25 ± 0.6 mL/min, Table 3) for the whole patient group.

**Table 3:** Weekly removed mass by dialysis and equivalent continuous clearance (ECC) of total, ionized and complexed calcium for all patients and separately for patients with removal and for patients with absorption of total calcium during weekly dialysis cycle. Negative sign of mass removed and ECC means absorption to the body from dialysis fluid, mean ± SD.

	Total	Ionized	Complexed^a^
*All patients*, *N = 25*			
Removed mass, mmol	5.67 ± 13.49	-13.53 ± 12.17	19.19 ± 14.14
ECC, mL/min	0.25 ± 0.60	-1.19 ± 1.11	5.15 ± 3.79
*Patients with weekly removal of calcium*, *N = 17*			
Removed mass, mmol	12.79 ± 8.71	-11.51 ± 10.41	24.3 ± 11.87
ECC, mL/min	0.56 ± 0.38	-1.00 ± 0.89	6.52 ± 3.18
*Patients with weekly absorption of calcium*, *N = 8*			
Removed mass, mmol	-9.48 ± 8.07*	-17.81 ± 15.12	8.33 ± 12.85*
ECC, mL/min	-0.42 ± 0.36*	-1.61 ± 1.47	2.23 ± 3.45*

* statistically significant difference vs. patient with weekly removal of calcium from body.

^a^ the removed mass for complexed calcium was measured in dialyzer, but its ECC was only estimated based on the average physiological value of complexed calcium in plasma for healthy individuals [17].

The weekly mass balance of total and complexed calcium *in dialysate* was on average positive, i.e., the gain of calcium in dialysate and its removal from the body by dialysis occurred, Table 3. The typical pattern was the absorption of ionized calcium from dialysis fluid and the removal of complexed calcium to dialysis fluid, Table 3. However, the net weekly calcium removal was effective only in 17 patients, and calcium was absorbed from dialysis fluid to the body in 8 patients. The patients with absorption of calcium had higher absorption of total calcium and lower removal of complexed calcium than the other patients, Table 3. High inter-patient variation in the removed calcium mass was observed: from -19.2 to 31.0 mmol for total calcium, from -36.8 to 5.1 mmol for ionized calcium, and from -14.9 to 61.1 mmol for complexed calcium per week.

**Fig 1:**
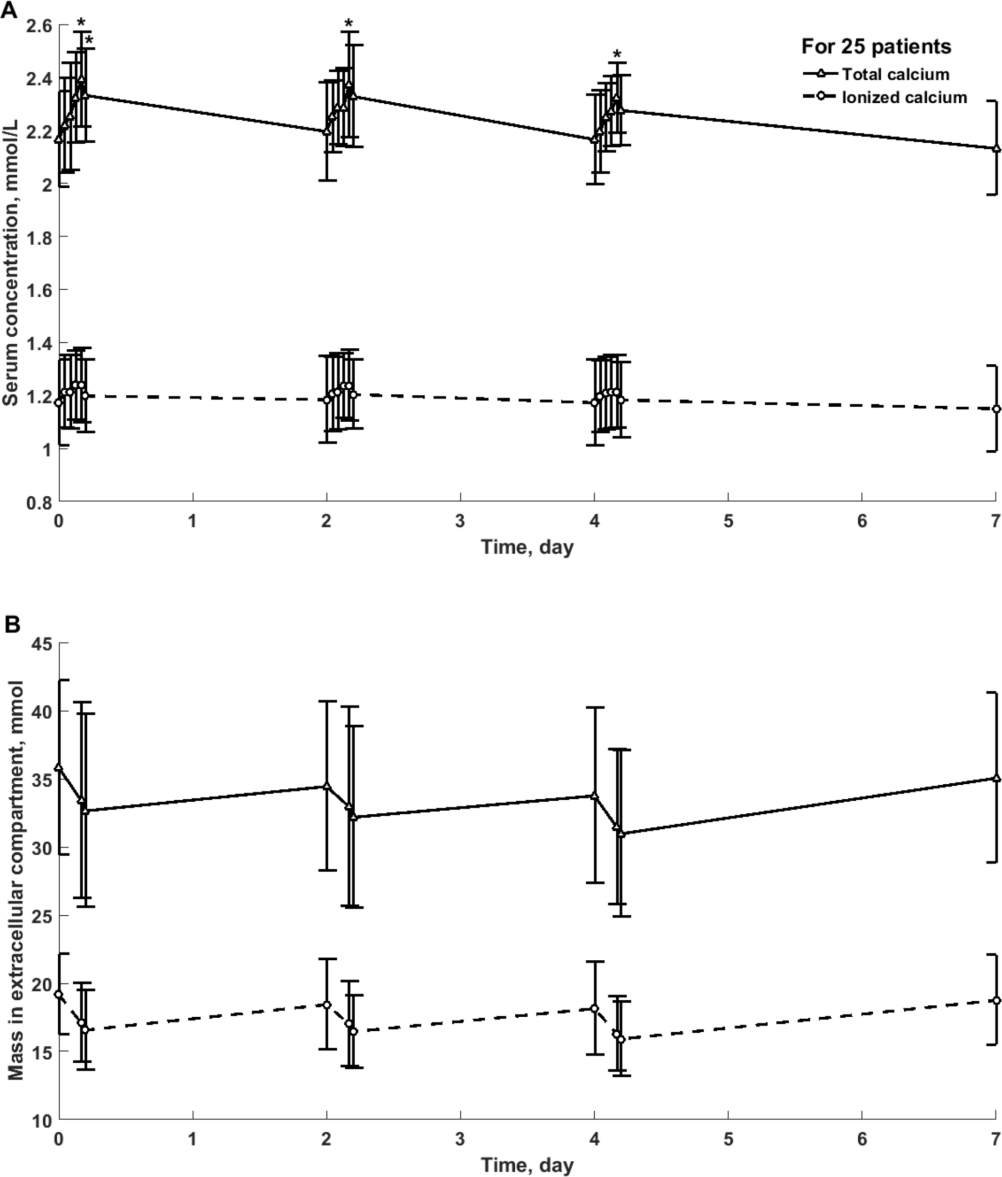
**Total and ionized calcium concentration in serum (panel A) and mass in extracellular compartment (panel B) in 25 patients during weekly cycle of three hemodialysis sessions, mean ± SD.** *—statistically significant difference vs. the beginning of HD.

**Fig 2:**
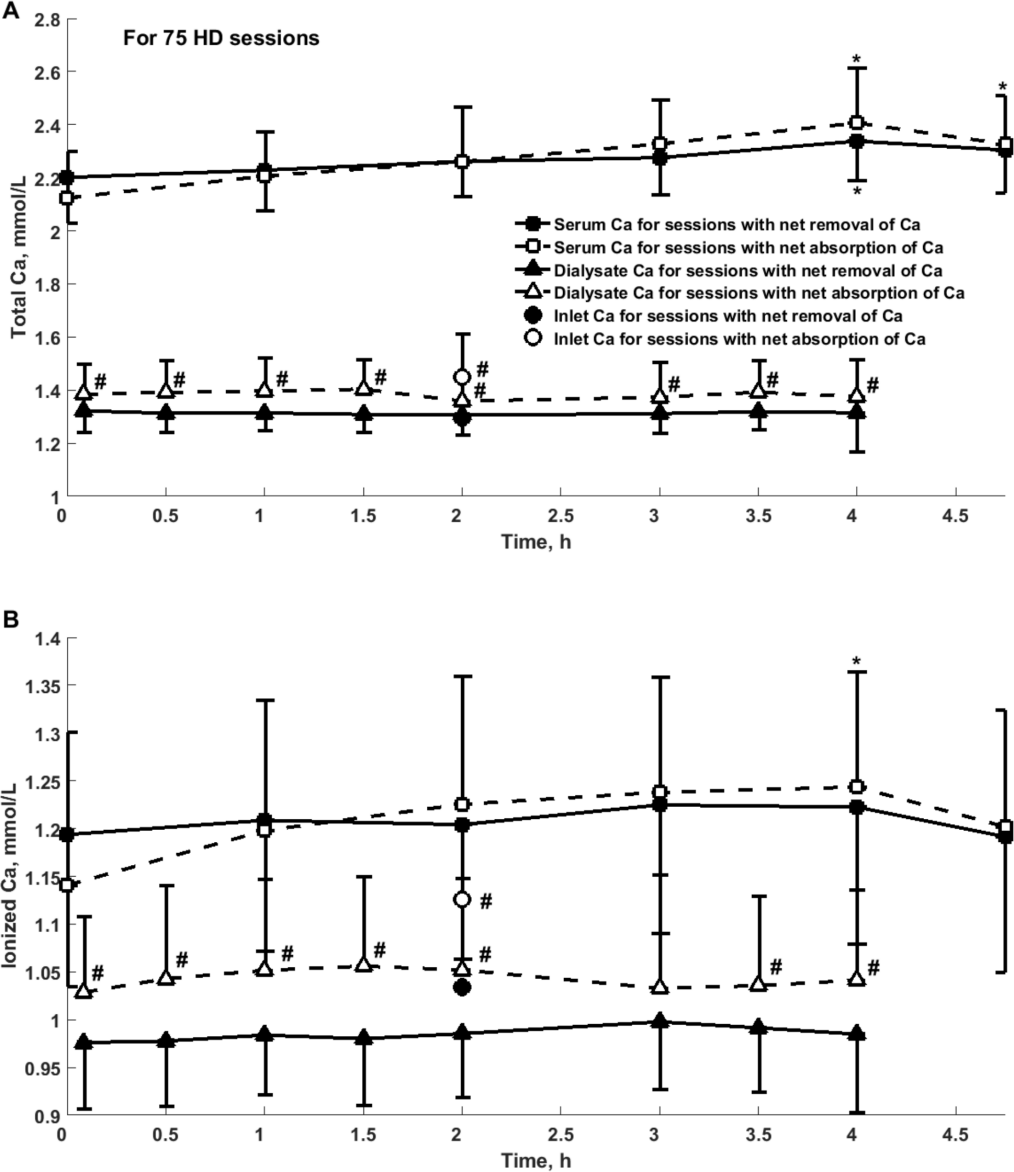
**Concentration of total (panel A) and ionized (panel B) calcium in serum, dialysate at the outlet and in dialysis fluid at the inlet of dialyzer during 75 hemodialysis sessions, mean +/- SD.** Concentration of total calcium (Panel A) and ionized calcium (Panel B) in serum (squares), dialysate at the outlet (triangles) and in dialysis fluid at the inlet of dialyzer (circles) during sessions with removal (filled markers, N = 50) or absorption (empty markers, N = 25) of total calcium; *—significant difference vs. the beginning of the session; #—significant difference vs. sessions with net removal of total calcium.

Among 75 sessions in 25 patients only in 48% of patients their sessions have uniform character (all three sessions had positive or negative mass balance). In 52% of patients negative and positive calcium mass balance occurred in individual sessions during one week cycle. Each patient used one type of dialyzer throughout three consecutive HD sessions. The type of dialyzer had no impact on the weekly calcium mass balance. Fig 1 presents the weekly profile of total and ionized calcium concentration and mass in 25 patients, whereas Fig 2 shows calcium concentration in serum and dialysate in 75 sessions divided into those with net removal (N = 50) and absorption (N = 25) of total calcium. Calcium concentration in serum for sessions with net absorption was not different in comparison with sessions with net removal of calcium, whereas concentration in output dialysate was different for these two types of sessions with negative and positive mass balance, Fig 2.

The concentration of total calcium in serum increased during dialysis and slightly decreased at 45 min after the end of dialysis, Table 4, Figs 1 and 2. The differences were observed for all sessions with the most pronounced increase in concentration during sessions with absorption of calcium from dialysis fluid, Table 4, Figs 1 and 2. Although the concentration of ionic calcium in serum had similar pattern, the differences were not statistically significant with the exception of the sessions with net absorption of calcium, Table 5, Fig 2.

**Table 4:** Concentration and mass of total calcium in extracellular compartment before, after and 45 min after the end of HD during all sessions and for sessions after 3- and 2-day break, and separately for sessions with removal and sessions with absorption of total calcium by dialysis, mean ± SD.

Total Ca	Before HD	After HD	45 min after HD
*All sessions*, *N = 75*			
*Concentration*, *mmol/L*			
All sessions	2.18 ± 0.18	2.36 ± 0.17^#^	2.31 ± 0.17^#^
After 3-day break	2.17 ± 0.18	2.39 ± 0.18^#^	2.33 ± 0.18^#^
After 2-day break	2.18 ± 0.18	2.35 ± 0.17^#^	2.30 ± 0.16^#^
*Mass*, *mmol*			
All sessions	34.67 ± 6.33	32.6 ± 6.73	31.92 ± 6.57^#^
After 3-day break	35.81 ± 6.42	33.4 ± 7.17	32.64 ± 7.08
After 2-day break	34.09 ± 6.27	32.2 ± 6.54	31.57 ± 6.35
*Sessions with net removal of calcium*, *N = 50*			
*Concentration*, *mmol/L*			
All sessions	2.20 ± 0.17	2.34 ± 0.15^#^	2.30 ± 0.16
After 3-day break	2.21 ± 0.18	2.39 ± 0.17^#^	2.33 ± 0.17
After 2-day break	2.19 ± 0.17	2.30 ± 0.12^#^	2.29 ± 0.15
*Mass*, *mmol*			
All sessions	35.51 ± 5.84	32.50 ± 5.68^#^	31.98 ± 5.74^#^
After 3-day break	34.98 ± 6.30	31.42 ± 5.66	30.70 ± 5.92
After 2-day break	35.84 ± 5.62	33.16 ± 5.68	32.76 ± 5.58^#^
*Sessions with net absorption of calcium*, *N = 25*			
*Concentration*, *mmol/L*			
All sessions	2.12 ± 0.17	2.41 ± 0.21^#^	2.33 ± 0.18^#^
After 3-day break	2.02 ± 0.09	2.38 ± 0.21^#^	2.32 ± 0.19
After 2-day break	2.16 ± 0.18*	2.41 ± 0.21*^,^^#^	2.33 ± 0.18*
*Mass*, *mmol *			
All sessions	32.97 ± 7.03	32.81 ± 8.59	31.82 ± 8.11
After 3-day break	38.45 ± 6.65	39.68 ± 8.34	38.77 ± 7.40
After 2-day break	31.24 ± 6.36*	30.65 ± 7.64*	29.62 ± 7.16*

Statistically significant difference vs.

# the beginning of HD

* sessions after 3-day break.

**Table 5:** Concentration and mass of ionized calcium in extracellular compartment before, after and 45 min after the end of HD during all sessions and for sessions after 3- and 2-day break, and separately for sessions with removal and sessions with absorption of total calcium by dialysis, mean ± SD.

Ionized Ca	Before HD	After HD	45 min after HD
*All sessions*, *N = 75*			
*Concentration*, *mmol/L*			
All sessions	1.18 ± 0.16	1.23 ± 0.14	1.19 ± 0.14
After 3-day break	1.17 ± 0.16	1.24 ± 0.14	1.20 ± 0.14
After 2-day break	1.18 ± 0.16	1.23 ± 0.13	1.19 ± 0.13
*Mass*, *mmol*			
All sessions	18.58 ± 3.22	16.8 ± 2.90^#^	16.31 ± 2.75^#^
After 3-day break	19.17 ± 2.96	17.09 ± 2.90	16.57 ± 2.91^#^
After 2-day break	18.29 ± 3.33	16.66 ± 2.92^#^	16.18 ± 2.69^#^
*Sessions with net removal of calcium*, *N = 50*			
*Concentration*, *mmol/L*			
All sessions	1.19 ± 0.16	1.22 ± 0.14	1.19 ± 0.14
After 3-day break	1.21 ± 0.16	1.25 ± 0.15	1.21 ± 0.14
After 2-day break	1.18 ± 0.16	1.21 ± 0.14	1.18 ± 0.14
*Mass*, *mmol*			
All sessions	19.10 ± 2.89	16.82 ± 2.48^#^	16.36 ± 2.46^#^
After 3-day break	18.99 ± 3.12	16.24 ± 2.44^#^	15.73 ± 2.53^#^
After 2-day break	19.16 ± 2.80	17.18 ± 2.47^#^	16.75 ± 2.37^#^
*Sessions with net absorption of calcium*, *N = 25*			
*Concentration*, *mmol/L*			
All sessions	1.14 ± 0.16	1.24 ± 0.12^#^	1.20 ± 0.12
After 3-day break	1.05 ± 0.10	1.20 ± 0.12^#^	1.16 ± 0.13
After 2-day break	1.17 ± 0.17	1.26 ± 0.12*	1.21 ± 0.12*
*Mass*, *mmol *			
All sessions	17.55 ± 3.63	16.77 ± 3.67	16.20 ± 3.31
After 3-day break	19.75 ± 2.57	19.81 ± 2.73	19.22 ± 2.53
After 2-day break	16.86 ± 3.69*	15.80 ± 3.45*	15.24 ± 2.97*

Statistically significant difference vs.

# the beginning of HD

* sessions after 3-day break.

The mass of total calcium in extracellular compartment decreased during dialysis and later on after the session with the most pronounced change for the sessions with net removal of calcium from the body to dialysis fluid, Table 4. The decrease of ionic calcium mass in extracellular compartment was found in sessions with net removal of calcium but not for the sessions with net absorption, Table 5. However, in spite of the absorption of 9.48 mmol of calcium per week from dialysis fluid in 8 patients, Table 3, the mass of ionic calcium in extracellular compartment in these patients did not increased, probably because of the storage of the excess of calcium in the internal compartments [17, 25–27].

The net decrease in total calcium mass in extracellular compartment of 2.06 ± 2.87 mmol during dialysis, was similar to total calcium mass removed in dialyzer of 1.89 ± 6.32 mmol (Table 2, NS) and correlated to this mass with rho = 0.36, p < 0.001, and to the mass of ionic calcium absorbed from dialyzer (rho = 0.55, p < 0.001). The change of calcium mass in extracellular fluid was caused, at least in part, by the decrease in extracellular fluid volume by 2.18 ± 0.85 L per session as measured by bioimpedance, which was similar to the ultrafiltration volume of 2.45 ± 0.86 L per session (NS).

## Discussion

The net dialytic mass balance of calcium in the group of 25 patients dialyzed with calcium concentration 1.35 mmol/L was negative, i.e., calcium was removed from the body to dialysate, Table 2. Nevertheless, in agreement with previous reports for different calcium concentration in dialysis fluid [10–13, 16, 28], one third of our patients had positive mass balance—calcium was absorbed from dialysis fluid, Tables 3 and 4. The typical transport pattern was the absorption of ionic calcium from dialysis fluid and the removal of complexed calcium to dialysate (Tables 2 and 3) according to the concentration gradients of these two forms of calcium, Fig 2.

The decrease of calcium (total and ionic) mass in the extracellular compartment during dialysis was significant in the dialysis sessions with net removal of calcium to dialysis fluid mostly due to the reduced volume of extracellular fluid with no change in the concentration of ionic calcium in serum (Tables 4 and 5). In contrast, the calcium concentration in serum increased during the sessions with absorption of calcium from dialysis fluid, and, in spite of decreased extracellular volume, no change in the masses of total and ionic calcium was found (Tables 4 and 5). The increase in the concentration of total calcium in serum was observed in all dialysis sessions, probably because of the hemoconcentration of serum proteins (and therefore also protein bound calcium) due to ultrafiltration.

The estimation of total calcium mass in extracellular compartment should be considered approximate, because of the unknown concentration of total calcium in interstitial fluid. Although ionic and complexed calcium may be expected in equilibrium between serum and interstitial fluid, the protein bound calcium may have different concentration between these two compartments. Thus, our estimation of total calcium mass in extracellular compartment based on its concentration in serum may be considered as the upper limit for the real value.

Equivalent continuous clearance (ECC or EKR) was initially proposed as a tool alternative to urea KT/V for the analysis of the adequacy of HD [24]. It was also applied for creatinine and beta2-microglobulin, and its theoretical features as higher sensitivity to time and frequency of dialysis sessions than KT/V are well known; therefore it can be applied for the comparison of different dialysis modalities and schedules [29–32]. An important feature of ECC is its independence of the solute generation rate, which makes it an index that can replace KT/V in the evaluation of the adequacy of solute removal [33]. ECC can also be used for metabolically unstable patients, for example during acute renal disease [34, 35]. Furthermore, ECC evaluation is based only on the solute mass removed and solute concentration in plasma, and does not need the definition and assessment of the distribution volume for the solute. This feature may be especially useful for the assessment of the removal of solutes without well defined distribution compartment and sophisticated control of transport within the body, as recently demonstrated for phosphate [20, 21]. Therefore, the quantification of calcium removal/absorption during dialysis session with ECC seems to be an interesting approach for clinical studies.

The occurrence of three different forms of calcium in plasma complicates its ECC assessment and interpretation. Only two forms of calcium are transported across dialyzer membrane: ionized and complexed. Whereas the ionized calcium can be currently measured with good accuracy in plasma and dialysate, the estimation of complexed calcium in dialysate can be performed indirectly, as the difference of total and ionized calcium measured directly because of the absence of protein bound calcium in dialysate. Unfortunately, the estimation of complexed calcium in plasma can be performed only after measuring a few other ions beside ionized and total calcium [18], which is not typically performed in clinical studies. Therefore, our estimation of ECC for complexed calcium, Table 3, should be considered as tentative.

Another problem with calcium ECC is its dependence on calcium concentration in dialysis fluid, as the definition of ECC involves only the normalization to calcium plasma concentration, as it is typical for clearance [36], whereas the diffusive transport of calcium in dialyzer is actually proportional to the difference of calcium concentration in plasma and in dialysis fluid [5]. However, ECC is based on the total mass removed, i.e., removed both by diffusion and convection [20]. The variable direction of the calcium transport in dialyzer, which depends on the sign of calcium concentration difference, results sometimes in negative ECC values that should be interpreted as absorption of calcium from dialysis fluid to blood, Table 3. With all these complications and restrictions, calcium ECC can be a useful and simple measure of the efficiency of dialytic calcium transfer.

Beyond the scope of our study is to propose the optimal settings for dialysis regarding calcium, but due to the detailed clinical protocol (frequent sampling of calcium concentration in serum and dialysate as well as measurement of volumes of body compartments) we were able to present quantitatively the calcium transport during 75 single dialysis sessions and collectively in 25 patients for the weekly treatment. The analysis includes the calcium shift in dialyzer and changes of calcium concentration and mass within extracellular compartment. We analyzed total, ionized and complexed calcium and distinguished two types of transport (diffusion and convection). We compared sessions with net removal of calcium with those with net absorption of calcium from dialysis fluid. The application of equivalent continuous clearance, ECC, as a measure of dialysis efficiency was tested and analyzed. The individualization of dialysate calcium can be made according to the predialysis serum calcium level [37]. Preferably total and complexed calcium should be measured. An adequate calcium setting for dialysis should be the proper compromise between the need to guarantee cardiovascular stability during HD, the goal to maintain normal serum concentration and aimed to avoid the risk of calcium overload.

We conclude that total calcium is removed by dialysis on average six times slower than by the normal kidneys as estimated by *equivalent continuous clearance*, *ECC*, in HD patients on dialysis fluid of 1.35 mmol/L calcium. One third of our patients is on positive dialytic calcium mass balance due to absorption of calcium from dialysis fluid. The high variation in calcium mass balance in dialyzer is present. Whereas total calcium tends to be removed on average, it is mostly in the complexed form, whereas the absorption of free calcium ions dominates in most dialysis sessions. Diffusion is the prevalent mechanism of transport of both ion and complexed forms of calcium. The concentrations of total calcium in the extracellular compartment increases during dialysis sessions and decreases between the sessions, whereas the mass of total and ionic calcium decreases during the sessions in patients with net removal of calcium during dialysis. The details of calcium transport and mass balance in extracellular compartment during one week cycle of three HD sessions presented in our study facilitate the quantitative understanding of calcium kinetics in dialyzed patients.
