# Correction: Quantification of Dialytic Removal and Extracellular Calcium Mass Balance during a Weekly Cycle of Hemodialysis

**DOI:** 10.1371/journal.pone.0193604

**Published:** 2018-02-23

**Authors:** Jacek Waniewski, Malgorzata Debowska, Alicja Wojcik-Zaluska, Andrzej Ksiazek, Wojciech Zaluska

Due to two errors described below, several corrections to the text, figures and tables of the published paper are needed.

The first error occurred in the identification of the mode of ion selective electrode that was applied for the measurements of calcium ion concentration as the *indirect mode* instead of the *direct mode*. The selection of the mode is important only for the measurements in plasma because of the presence of charged protein.

The second error occurred in the formula for the correction of calcium ion concentration in plasma and resulted in the overestimation of the real value of this concentration. Actually, the appropriate formula for this correction yields only negligible change (less than 2%) in the measured values and therefore we have decided not to use any correction in the updated values. Therefore, the calcium ion concentrations in plasma and in dialysis fluid are now presented as measured by ion selective electrode in the direct mode.

In the “Patients and clinical data” subsection of the Methods section, the sixth sentence of the second paragraph should read: Concentrations of total (by calorimetric method with Arsenazo, Advia 1800, Siemens) and ionized (by ion selective electrode and potentiometry direct mode, RapidLab 348, Siemens) calcium were measured in serum before, at 1, 2 and 3 h, at the end and 45 min after each session, before the fourth dialysis session and in the outlet dialysate every 0.5 h.

In the “Calculations” subsection of the Methods section, the first sentence of the third paragraph should be removed.

Due to the errors described, the published Fig 1, Fig 2, Table 3, and Table 5 are incorrect. Please see the corrected versions here.

**Fig 1:**
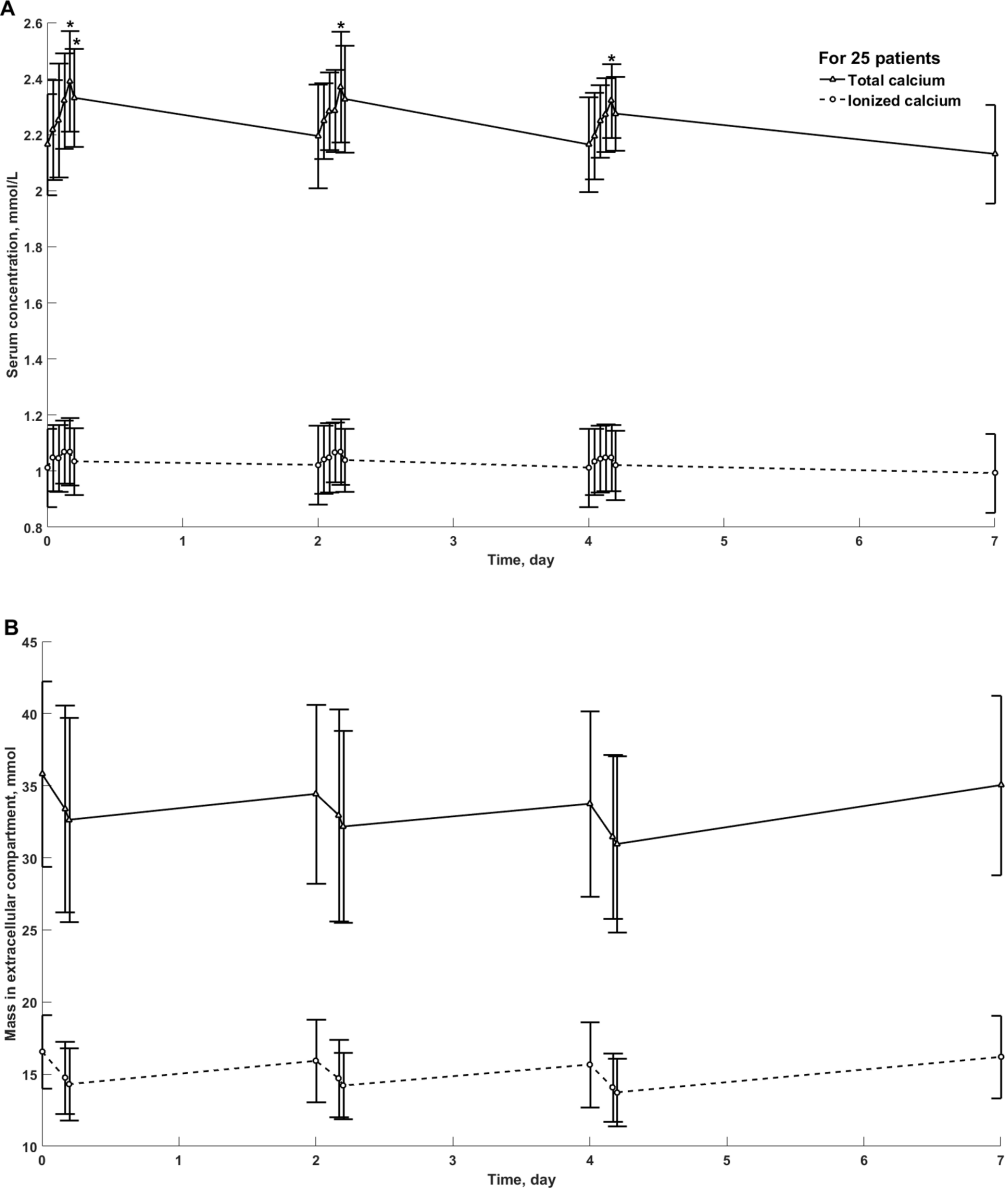
**Total and ionized calcium concentration in serum (panel A) and mass in extracellular compartment (panel B) in 25 patients during weekly cycle of three hemodialysis sessions, mean ± SD.** *—statistically significant difference vs. the beginning of HD.

**Fig 2:**
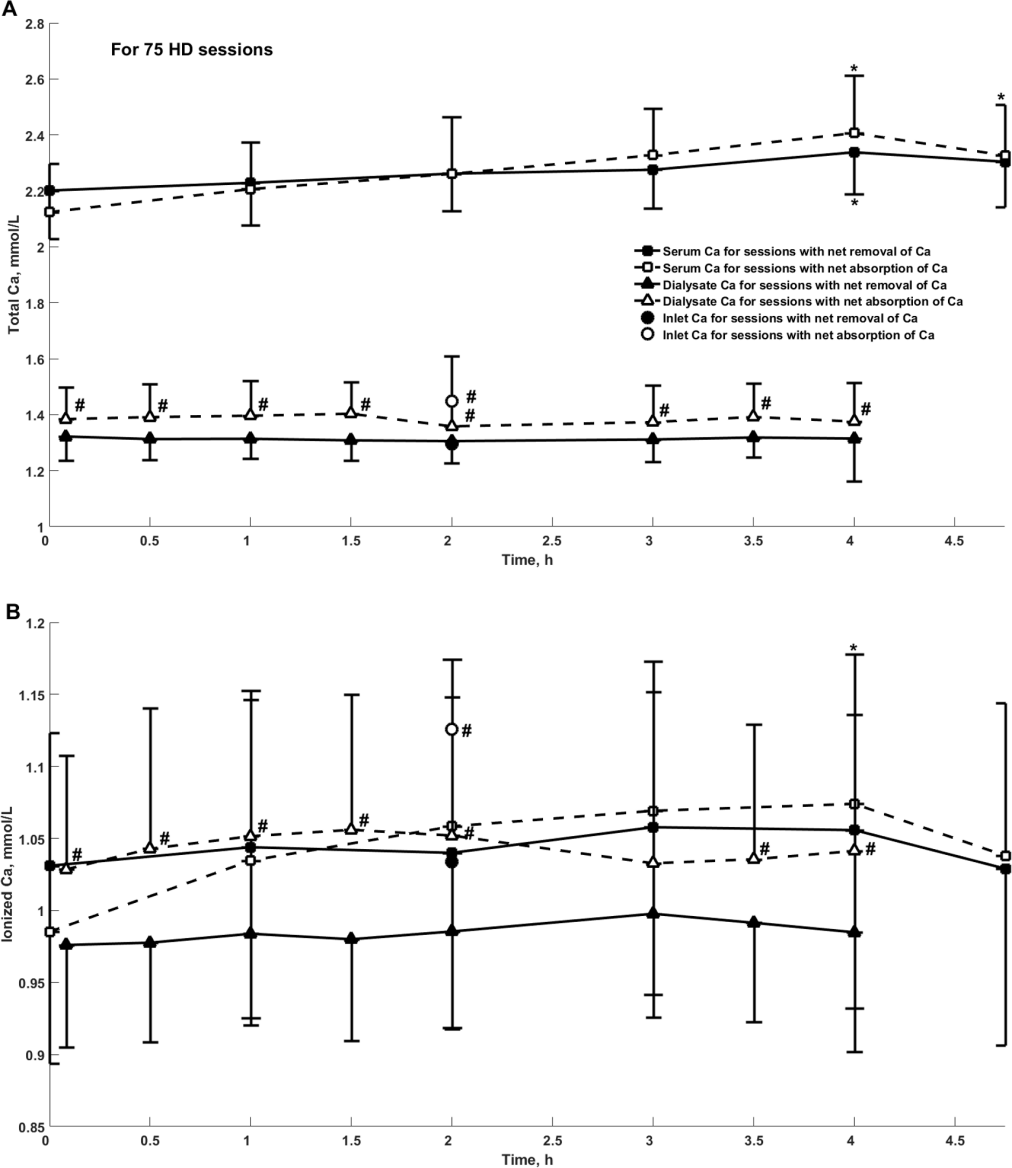
**Concentration of total (panel A) and ionized (panel B) calcium in serum, dialysate at the outlet and in dialysis fluid at the inlet of dialyzer during 75 hemodialysis sessions, mean +/- SD.** Concentration of total calcium (Panel A) and ionized calcium (Panel B) in serum (squares), dialysate at the outlet (triangles) and in dialysis fluid at the inlet of dialyzer (circles) during sessions with removal (filled markers, N = 50) or absorption (empty markers, N = 25) of total calcium; *—significant difference vs. the beginning of the session; #—significant difference vs. sessions with net removal of total calcium.

**Table 3:** Weekly removed mass by dialysis and equivalent continuous clearance (ECC) of total, ionized and complexed calcium for all patients and separately for patients with removal and for patients with absorption of total calcium during weekly dialysis cycle. Negative sign of mass removed and ECC means absorption to the body from dialysis fluid, mean ± SD.

	Total	Ionized	Complexed^a^
*All patients*, *N = 25*			
Removed mass, mmol	5.67 ± 13.49	-13.53 ± 12.17	19.19 ± 14.14
ECC, mL/min	0.25 ± 0.60	-1.38 ± 1.29	5.15 ± 3.79
*Patients with weekly removal of calcium*, *N = 17*			
Removed mass, mmol	12.79 ± 8.71	-11.51 ± 10.41	24.3 ± 11.87
ECC, mL/min	0.56 ± 0.38	-1.16 ± 1.03	6.52 ± 3.18
*Patients with weekly absorption of calcium*, *N = 8*			
Removed mass, mmol	-9.48 ± 8.07*	-17.81 ± 15.12	8.33 ± 12.85*
ECC, mL/min	-0.42 ± 0.36*	-1.87 ± 1.70	2.23 ± 3.45*

* statistically significant difference vs. patient with weekly removal of calcium from body

^a^ the removed mass for complexed calcium was measured in dialyzer, but its ECC was only estimated based on the average physiological value of complexed calcium in plasma for healthy individuals [17].

**Table 5:** Concentration and mass of ionized calcium in extracellular compartment before, after and 45 min after the end of HD during all sessions and for sessions after 3- and 2-day break, and separately for sessions with removal and sessions with absorption of total calcium by dialysis, mean ± SD.

Ionized Ca	Before HD	After HD	45 min after HD
*All sessions*, *N = 75*			
*Concentration*, *mmol/L*			
All sessions	1.02 ± 0.14	1.06 ± 0.12	1.03 ± 0.12
After 3-day break	1.01 ± 0.14	1.07 ± 0.12	1.03 ± 0.12
After 2-day break	1.02 ± 0.14	1.06 ± 0.12	1.03 ± 0.12
*Mass*, *mmol*			
All sessions	16.05 ± 2.78	14.51 ± 2.51^#^	14.09 ± 2.38^#^
After 3-day break	16.56 ± 2.56	14.76 ± 2.51	14.31 ± 2.51^#^
After 2-day break	15.79 ± 2.87	14.39 ± 2.53^#^	13.97 ± 2.32^#^
*Sessions with net removal of calcium*, *N = 50*			
*Concentration*, *mmol/L*			
All sessions	1.03 ± 0.14	1.06 ± 0.12	1.03 ± 0.12
After 3-day break	1.05 ± 0.14	1.08 ± 0.13	1.04 ± 0.12
After 2-day break	1.02 ± 0.14	1.04 ± 0.12	1.02 ± 0.12
*Mass*, *mmol*			
All sessions	16.49 ± 2.50	14.53 ± 2.14^#^	14.13 ± 2.12^#^
After 3-day break	16.40 ± 2.70	14.02 ± 2.11^#^	13.58 ± 2.19^#^
After 2-day break	16.55 ± 2.41	14.84 ± 2.13^#^	14.47 ± 2.05^#^
*Sessions with net absorption of calcium*, *N = 25*			
*Concentration*, *mmol/L*			
All sessions	0.99 ± 0.14	1.07 ± 0.10^#^	1.04 ± 0.11
After 3-day break	0.90 ± 0.09	1.04 ± 0.10^#^	1.01 ± 0.11
After 2-day break	1.01 ± 0.14	1.09 ± 0.11*	1.05 ± 0.11*
*Mass*, *mmol *			
All sessions	15.16 ± 3.14	14.48 ± 3.17	13.99 ± 2.86
After 3-day break	17.06 ± 2.22	17.11 ± 2.35	16.61 ± 2.19
After 2-day break	14.56 ± 3.19*	13.65 ± 2.98*	13.16 ± 2.56*

Statistically significant difference vs.

# the beginning of HD

* sessions after 3-day break.
